# Patterns of Mandibular Fractures and Associated Comorbidities in Peshawar, Khyber Pakhtunkhwa

**DOI:** 10.7759/cureus.5753

**Published:** 2019-09-25

**Authors:** Sahd Rashid, Jawad A Kundi, Amna Sarfaraz, Asif U Qureshi, Adnan Khan

**Affiliations:** 1 Oral and Maxillofacial Surgery, Rehman Medical Institute, Peshawar, PAK; 2 Oral and Maxillofacial Surgery, Sardar Begum Dental College, Peshawar, PAK; 3 Dentistry, Rehman College of Dentistry, Peshawar, PAK; 4 Oral and Maxillofacial Surgery, Rehman College of Dentistry, Peshawar, PAK; 5 Department of Pediatrics, Rehman Medical Institute, Peshawar, PAK

**Keywords:** mandible fracture, rta, parasymphysis

## Abstract

Objectives

The objective of this study was to assess patterns of mandibular fractures and associated comorbidities in Peshawar, Pakistan.

Methodology

This multicenter, descriptive, cross-sectional study analyzed patients aged >15 years who had been clinically or radiographically diagnosed with mandibular fractures from January to December, 2015. Patients with pathological fractures or bomb-blast injuries were excluded. Data were analyzed using IBM SPSS Statistics for Windows, Version 20.0 (IBM Corp., Armonk, NY).

Results

The 138 patients diagnosed with mandibular fractures in 2015 included 108 men (78.3%) and 30 women (21.7%), with a male preponderance of 3.6:1. Most patients (56%) were aged 15-25 years, followed by those aged 26-35 years (26%). The most frequent cause of fractures was road traffic accidents (RTAs; 59.42%), followed by falls (18.8%). RTAs were predominant in men (89%); whereas, falls were predominant in women (80%). Fractures due to firearm injuries and interpersonal violence were more frequent in men (p <0.001). In patients with unilateral fractures, the most common fracture site was the parasymphysis (24.6%) followed by the symphysis (10.1%). In patients with bilateral fractures, the most common fracture sites were the parasymphysis and condyle (11.6%), followed by the parasymphysis and angle (8.0%).

Conclusions

RTA was the most frequent cause of mandibular fracture and trauma. Mandibular fractures were more common in men than women, with most patients aged 15-25 years. The most common fracture site was the parasymphysis.

## Introduction

The facial area is one of the most common sites of injury [1-2]. The mandible is fractured more frequently than any other facial bone, likely because it is exposed and protruding [1,3]. In addition to functional loss, a mandibular fracture can result in mild to moderate impairment or defect [4].

Mandibular fractures are the most frequent type of fracture in the maxillofacial region [5]. Mandibular fractures may occur alone or together with other facial bone fractures. The predicted ratio of mandible to zygomatic to maxillary bone fractures in patients experiencing maxillofacial injury is 9:4:1 [5].

The mandible is particularly prone to maxillofacial trauma because of its unique shape, mobility, and prominence in the facial skeleton. It is the second most common facial bone experiencing traumatic injuries, accounting for 15.5%-59% of all facial fractures [6]. Patients with a broken lower jaw experience pain, difficulty chewing and talking, and esthetic disfigurement [7]. These injuries are often accompanied by psychological effects, along with significant financial costs [7].

The epidemiology of mandible fractures varies over time and in different countries. The etiology of these fractures is multifactorial, with the type and frequency of fracture dependent on socioeconomic status, culture, technology, demography, and economic factors. 

No recent comprehensive study has assessed the significance of mandibular fracture in South Asian countries. Therefore, this study analyzed patterns of mandibular fractures and associated comorbidities in patients evaluated at departments of maxillofacial surgery at teaching hospitals in Peshawar, Pakistan. Determining the frequency of mandibular fractions and their patterns of occurrence can enhance early diagnosis and treatment, thereby reducing morbidity and mortality associated with these fractures.

## Materials and methods

The present study was carried out at the departments of oral and maxillofacial surgery of three hospitals in Peshawar, Pakistan: Sardar Begum Dental College and Hospital, Rehman Medical Institute, and Northwest General Hospital. Patients aged >15 years presenting at these hospitals with mandibular fractures, including X-rays showing a discontinuity of the mandible, from January to December 2015, were included. Patients with pathological fractures or fractures due to bomb blast injuries were excluded.

Based on the World Health Organization software for sample size determination, the minimum sample size was determined to be 138 patients, with a 95% confidence level and an 8% margin of error. Non-probability consecutive sampling technique was used as the sampling method.

Patients were examined clinically and radiographically in the outpatient departments of the three hospitals, and a detailed history was taken.

The study protocol was approved by the Ethical Committee of Gandhara University, Peshawar, and all included patients provided written informed consent. Patient information, including, name, age, and gender were recorded on pre-designed forms. Data were analyzed using IBM SPSS Statistics for Windows, Version 20.0 (IBM Corp., Armonk, NY). Continuous variables were reported as mean and standard deviation and categorical variables as number (percent). Patients were stratified by age, gender, and site of mandibular fracture to determine the etiology of mandibular fractures. The level of significance was set at P < 0.05.

## Results

**Figure 1:**
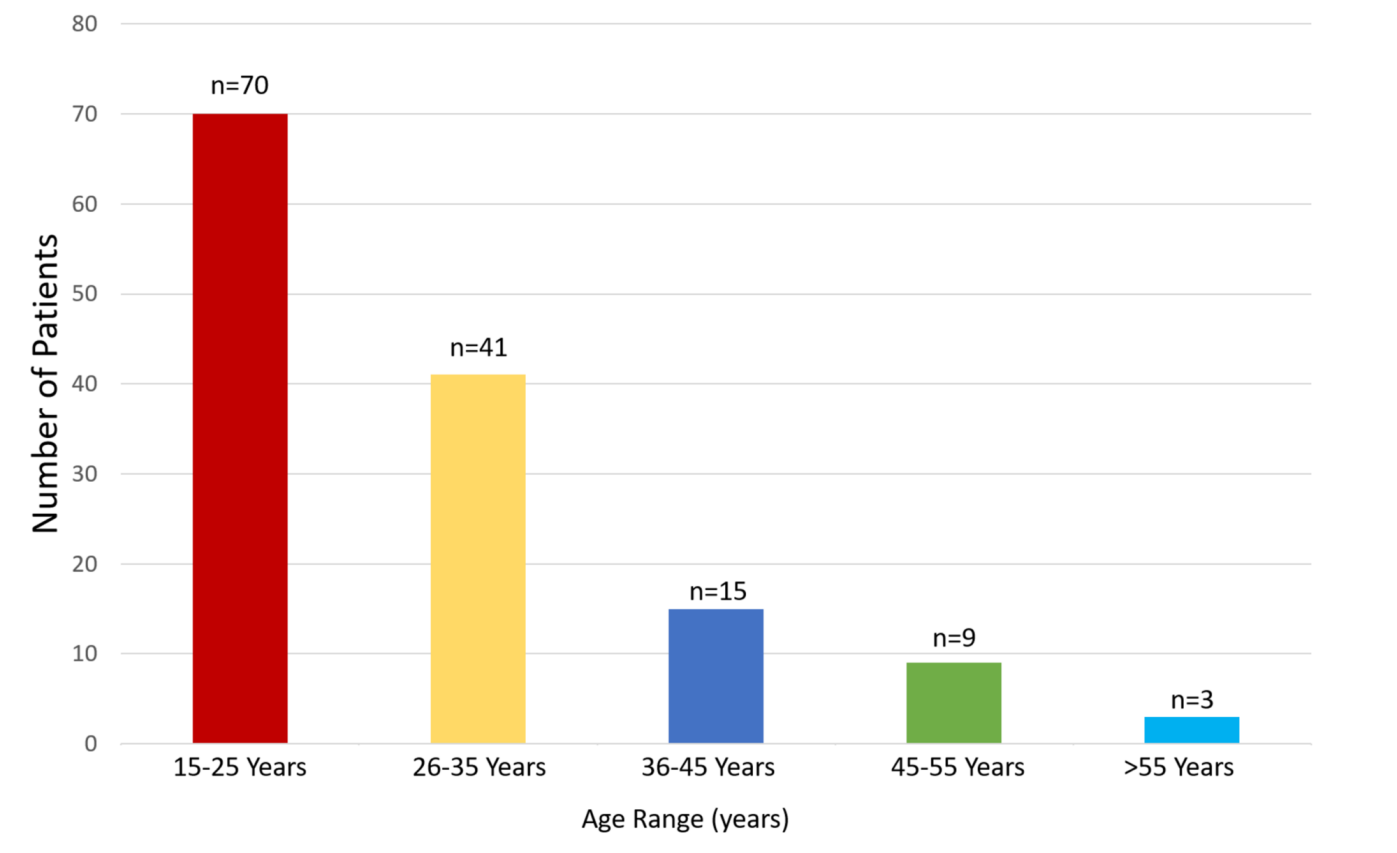
Age distribution of patients affected by mandibular fractures (n=138)

The 138 patients included 108 young men (78.3%) and 30 young women (21.7%), resulting in a male-to-female preponderance of 3.6:1. The majority (56%) were aged 15-25 years, with 26% aged 26-35 years, indicating that young adults are mostly affected by mandibular fractures (Figure 1). 

**Figure 2:**
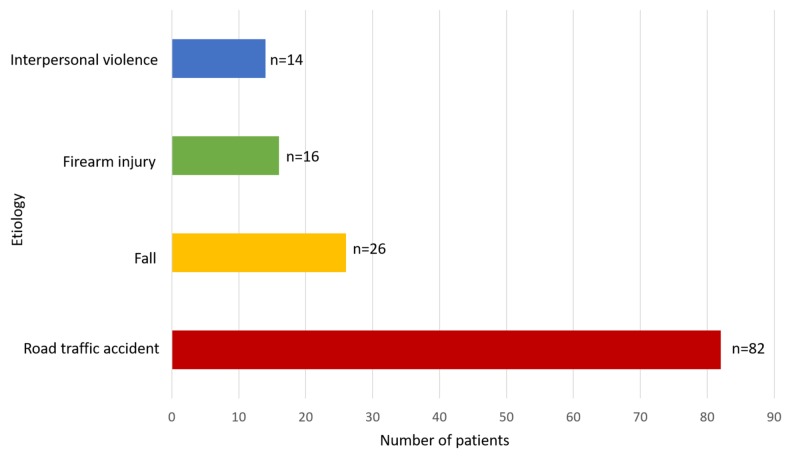
Etiology of mandibular fractures (n=138)

The most frequent cause of mandibular fracture was road traffic accidents (RTAs; 59.4%), followed by falls (18.8%) (Figure 2). RTAs were predominant in men (89%); whereas, falls were predominant in women (80%). Fractures due to firearm injuries and interpersonal violence were more frequent in men (p <0.001). 

In patients with unilateral fractures, the most frequent site was the parasymphysis (24.6%), followed by the symphysis (10.1%). Other sites included the condyle (8.7%), body (8.7%), dentoalveolar area (6.5%), angle (2.2%), ramus (2.2%), and comminuted fractures (1.4%) (Table 1). In patients with bilateral fractures, the most common sites were the parasymphysis and condyle (11.6%), followed by the parasymphysis and angle (8.0%), condyle and body (4.3%), and dentoalveolar area and angle (3.6%).

**Table 1:** Distribution of anatomic sites of mandibular fractures (n=138)

Sites	Number	Percentage
Unilateral fractures		
Parasymphysis	34	24.63%
Symphysis	14	10.14%
Condyle	12	8.69%
Body	12	8.69%
Dentoalveolar	9	6.52%
Angle	3	2.17%
Ramus	3	2.17%
Comminuted fracture	2	1.44%
Bilateral fractures		
Parasymphysis + condyle	16	11.59%
Parasymphysis + angle	11	7.97%
Condyle + body	6	4.34%
Dentoalveolar + angle	5	3.62%
Body + symphysis	4	2.89%
Condyle + Dentoalveolar	4	2.89%
Left body + Right angle	2	1.44%
Angle + Dentoalveolar + LeFort 2.	1	0.72%

Table 2 shows the associated comorbidities in the study population. 

**Table 2:** Comorbidities observed in patients with mandibular fractures (n=138) TMJ: temporomandibular joint.

Comorbidities	Number	Percentage
Hard Tissue Injury		
LeFort fracture	35	25.36%
Zygomatic fracture	28	20.28%
Fracture of the extremities	15	10.86%
c-spine injury	15	10.86%
Clavicle injury	15	10.86%
TMJ dislocation	15	10.86%
Nasal fracture	15	10.86%
Soft Tissue Injury		
Facial laceration	62	44.92%
Swelling	35	25.36%
Epistaxis	17	12.31%
Ocular injury	14	10.8%
Hyphema + retrobulbar injury	3	2.17%
Infection	2	1.44%
Multiple wounds	2	1.44%

## Discussion

Epidemiological surveys have shown that the causes, incidence, and patterns of mandibular fracture vary by geographical region, socioeconomic condition, cultural characteristic, and era. In the present study, 56% of patients with mandibular fracture were aged 15-25 years, consistent with the results of previous studies [8-9], but differs from that in a Jordanian population [10]. Moreover, mandibular fracture was more common in men than in women, with a 3.6:1 ratio, consistent with previous findings [1,11-12]. People are more active during the second and third decades of life than during other decades, making them more vulnerable to trauma. Moreover, men participate in more outdoor activities than women.

Previous epidemiological studies have shown that RTA and falls are important causes of mandibular fracture in developing countries [13-14]. In developed countries, however, the leading causes of mandibular fracture are physical assault and interpersonal violence [7]. Seatbelt legislation markedly reduced the incidence of mandibular fracture due to RTA in developed countries, although the incidence was increased by the abuse of alcohol and the use of illicit drugs [15]. The high number of mandibular fractures attributed to RTA in Pakistan is likely due to the lack of seat belt laws, speeding and overloading of vehicles, underage driving, and poor conditions of roads and vehicles. The high incidence of mandibular fractures due to firearm injuries (12%) was likely due to tribal quarrels and mode of life in the Khyber Pakhtunkhwa (KPK) province, in which the possession of arms is a part of cultural life.

The most common site of mandibular fracture was the parasymphysis (35%) followed by the symphysis (14%) and condyle (11%) [16]. The causes and anatomic sites of mandibular fracture may correlate. For example, the majority of parasymphysis fractures were caused by RTA. The most common soft tissue comorbidity was facial laceration; whereas, the most common hard tissue comorbidity was LeFort fracture. Similar findings were reported by a study in India [17].

An awareness campaign to educate the public, especially drivers, about the importance of restraints and protective measures in motor vehicles should be started. Education of parents about the consequences of falls in children may reduce the incidence of injury in pediatric populations.

## Conclusions

Most patients who experienced mandibular fractures were young men. The most common etiological factor was RTA, followed by falls, whereas the most frequently fractured site was the parasymphysis. The incidence of RTAs and resultant fractures may be reduced by strict enforcement of seat belt laws, speed limits, and other traffic rules.
